# Comparative analysis of influenza healthcare disparities in the United States using retrospective administrative claims from Medicaid and commercial databases, 2015–2019

**DOI:** 10.1371/journal.pone.0321208

**Published:** 2025-05-22

**Authors:** Jennifer L. Matas, Kira Raskina, Sabine Tong, Derrick Forney, Bruno Scarpellini, Mario Cruz-Rivera, Gary Puckrein, Liou Xu

**Affiliations:** 1 Center for Clinical and Social Research, National Minority Quality Forum, Washington, District of Columbia, United States of America; 2 Real Word Evidence, Opella Healthcare, Barcelona, Spain; 3 Real World Evidence, Opella Healthcare, Neuilly-sur-Seine, France; 4 Real World Evidence, Opella Healthcare, São Paulo, SP, Brazil; 5 Global Switch Medical Science, Opella Healthcare, Morristown, New Jersey, United States of America; 6 Offices of the President, National Minority Quality Forum, Washington District of Columbia, United States of America; Neurocrine Biosciences Inc, UNITED STATES OF AMERICA

## Abstract

**Background:**

Influenza-related healthcare utilization among Medicaid patients and commercially insured patients is not well-understood. This study compared influenza-related healthcare utilization and assessed disease management among individuals diagnosed with influenza during the 2015–2019 influenza seasons.

**Methods:**

This retrospective cohort study identified influenza cases among adults (18–64 years) using data from the Transformed Medicaid Statistical Information System (T-MSIS) Analytic Files (TAF) Research Identifiable Files (RIF) and Optum’s de-identified Clinformatics® Data Mart Database (CDM). Influenza-related healthcare utilization rates were calculated per 100,000 patients by setting (outpatient, emergency department (ED), inpatient hospitalizations, and intensive care unit (ICU) admissions) and demographics (sex, race, and region). Rate ratios were computed to compare results from both databases. Influenza episode management assessment included the distribution of the index point-of-care, antiviral prescriptions, and laboratory tests obtained.

**Results:**

The Medicaid population had a higher representation of racial/ethnic minorities than the CDM population. In the Medicaid population, influenza-related visits in outpatient and ED settings were the most frequent forms of healthcare utilization, with similar rates of 652 and 637 visits per 100,000, respectively. In contrast, the CDM population predominantly utilized outpatient settings. Non-Hispanic Blacks and Hispanics exhibited the highest rates of influenza-related ED visits in both cohorts. In the Medicaid population, Black (64.5%) and Hispanic (51.6%) patients predominantly used the ED as their index point-of-care for influenza. Overall, a greater proportion of Medicaid beneficiaries (49.8%) did not fill any influenza antiviral prescription compared to the CDM population (37.0%).

**Conclusion:**

Addressing disparities in influenza-related healthcare utilization between Medicaid and CDM populations is crucial for equitable healthcare access. Targeted interventions are needed to improve primary care and antiviral access and reduce ED reliance, especially among racial/ethnic minorities and low-income populations.

## Introduction

Influenza, commonly known as the flu, is a highly contagious viral respiratory illness. It poses a significant public health challenge, leading to substantial morbidity and mortality worldwide, especially during seasonal outbreaks [1]. While the flu can occur year-round in the United States, flu activity is highest between December and February, coinciding with the fall and winter seasons. The Centers for Disease Control and Prevention (CDC) estimates that between the years 2010 and 2023, the flu has resulted in 9.3 million to 41 million illnesses per year, 100,000–710,000 hospitalizations per year, and 4,900–51,000 deaths annually [2]. Populations at highest risk for influenza hospitalization include individuals living in crowded conditions, those with lower incomes, individuals with chronic diseases, and those who harbor a high level of distrust toward the government and vaccinations [3–6].

Studies have shown that Black and Hispanic individuals are disproportionately affected by influenza. Health disparities related to influenza outcomes are well-documented, with minority populations experiencing higher rates of hospitalization and mortality compared to non-Hispanic White populations. During past influenza outbreaks, studies have shown that hospitalization rates for Black and Hispanic populations were 2–3 times higher compared to non-Hispanic White populations [7]. Various economic, social, and healthcare access barriers contribute to the disparities experienced by minority groups, who are often disproportionately affected by poverty, limited job opportunities, and inadequate access to healthcare [8,9].

Medicaid, the largest source of funding for medical and health-related services for low-income and disabled individuals in the US, plays a crucial role in serving low-income racial and ethnic minority populations, with approximately half of nonelderly Medicaid beneficiaries belonging to these groups [10]. Medicaid beneficiaries face specific challenges when accessing timely influenza treatment. These individuals often have limited access to primary care services and preventive care, which can result in delayed diagnosis and treatment of influenza [11,12]. Consequently, these barriers to primary care access may contribute to higher emergency department (ED) use among Medicaid enrollees than their privately insured counterparts [13]. This is further influenced by lower co-payments for ED visits and the perception of the ED as a convenient place to receive multiple services at once, which is especially appealing for patients with transportation issues [13,14].

In contrast, individuals with private insurance are 1.6 times more likely to secure primary care appointments and 3.3 times more likely to secure specialty care appointments than those with Medicaid [15]. Increased access to primary care enables individuals with commercial insurance to benefit from earlier diagnosis, timely treatment, prescription access, and preventive services, including vaccinations. Patients with commercial insurance have higher influenza vaccination rates compared to Medicaid recipients [16] which can reduce flu burden including hospitalization and ED visits. However, disparities in influenza-related healthcare utilization between commercial and Medicaid populations are not yet fully understood.

EDs often experience a surge in patient volume and resource strain during peak flu seasons, leading to longer wait times and challenges in providing timely and appropriate patient care. Some studies suggest that the expansion of rapid point-of-care tests could allow for appropriate use of influenza pharmacological treatments and decrease patient length of stay in the ED [17]. Additionally, early administration of flu antivirals such as oseltamivir phosphate (Tamiflu®, F. Hoffmann-La Roche Ltd; and generics) can reduce symptoms and lessen flu complications, working best when initiated within 1–2 days of symptom onset [18]. However, these antivirals are not available over the counter and must be prescribed by a provider [18].

Individuals with commercial insurance generally face fewer barriers to obtaining prescription medications. One study found that those with employer-based private insurance were 58% more likely to have a personal physician and 22% less likely to forgo medications due to cost compared to Medicaid beneficiaries [19]. While disparities in access to influenza antivirals between Medicaid and commercially insured populations have not been fully explored, evidence suggests gaps in access among Medicaid populations, particularly for African American patients. For example, a study in Georgia reported that Medicaid patients with disabilities received antivirals in only 14.5% of diagnosed influenza cases, with White patients being 2.9 times more likely to receive antiviral drugs compared to their African American counterparts [20].

This study evaluated and compared influenza-related healthcare utilization and disease management among individuals diagnosed with influenza during the 2015–2019 influenza seasons from the Medicaid and commercial insurance populations. This research utilized claims data from two sources: Medicaid and Optum’s de-identified Clinformatics® Data Mart Database (CDM). Claims data provide detailed information on healthcare encounters and treatments across large and diverse populations. While population-based surveys offer valuable insights into self-reported behaviors and experiences, claims data capture objective, longitudinal, and comprehensive records of healthcare utilization, including outpatient visits, hospitalizations, and prescription fills. Additionally, using claims data from both Medicaid and commercial insurance sources allowed us to examine disparities in influenza-related care across different socioeconomic groups and insurance types, addressing gaps in understanding healthcare access and outcomes in these populations.

## Methods

### Study design, data sources, and study periods

The two data sources used for this retrospective study were the US Medicaid claims data and CDM. Medicaid is a US federal and state-funded public insurance program that serves as the largest source of medical and health-related funding for low-income individuals including children, parents, pregnant individuals, seniors, and disabled persons [21,22]. As of January 2024, approximately 84 million individuals were enrolled in Medicaid across all 50 states and the District of Columbia [23]. Over half of Medicaid beneficiaries comprise of underrepresented racial and ethnic groups (18.6% Black, 29.2% Hispanic, 4.7% Asian/Native and Pacific Islander, and 0.9% American Indian/Alaska Native) [24]. The Transformed Medicaid Statistical Information System (T-MSIS) Analytic Files (TAF) Research Identifiable Files (RIF), referred to as TAF RIF were used. TAF RIF files include data on Medicaid and Children’s Health Insurance Program (CHIP) demographic and enrollment information as well as healthcare service utilization and payment claims [25].

CDM is derived from a database of administrative health claims for members of large commercial and Medicare Advantage health plans with additional socio-economic status variables. The database includes approximately 17–19 million annual covered individuals amounting to over 65 million people over a nine-year period (January 2007 through December 2021) [26]. CDM is statistically de-identified under the Expert Determination method consistent with the Health Insurance Portability and Accountability Act (HIPAA) and managed according to Optum customer data use agreements [27,28]. CDM administrative claims submitted for payment by providers and pharmacies are verified, adjudicated, and de-identified prior to inclusion. CDM claims data, including patient-level enrollment information, are derived from claims submitted for all medical and pharmacy healthcare services with information related to healthcare costs and resource utilization. The population is geographically diverse, spanning all 50 states and Washington, D.C. [26].

The analyses for this study combined Medicaid and CDM cohorts for the following four influenza seasons from 2015 to 2019. The Medicaid and CDM datasets were analyzed separately for the four influenza seasons from 2015 to 2019. Each analysis was conducted independently within its respective dataset. An influenza season was defined from October 1 through May 31 for the specific flu season under consideration (e.g., Oct 1, 2015 through May 31, 2016, for the 2015/2016 influenza season). For this retrospective study, de-identified data were accessed between September 2022 and August 2024 for research and analysis. Because the data is de-identified and cannot be linked to individual patients, Institutional Review Board (IRB) approval was not required. The data were analyzed anonymously therefore patient consent was not obtained.

### Study population

In the Medicaid study population, patients between 18 and 64 years of age were included if they had continuous coverage during the entire influenza season; however, discontinuation of coverage due to death was permitted. The same inclusion criteria were applied to the CDM database. In the CDM cohort, patients who were dual-eligible (Medicare and Medicaid) were excluded from the analyses. In both datasets, individuals younger than 18 years and those aged 65 years or older were excluded. No additional exclusions were applied based on specific conditions or incomplete data. An available case analysis approach was used, allowing patients to contribute to analyses for which their data were available while maximizing the utility of the dataset.

### Outcome measures

The primary outcomes of interest included the following influenza-related healthcare utilization endpoints: i) influenza-related outpatient visits, ii) ED visits, iii) inpatient hospitalizations, and iv) intensive care unit (ICU) admissions. These healthcare utilization endpoints were analyzed as count variables to assess the burden of influenza. Influenza-related healthcare utilization was defined by an International Classification of Diseases, 10th revision, Clinical Modification (ICD-10-CM) code for influenza (J09.xx-J11.xx) as the principal diagnosis for inpatient and ICU admissions, or as the first or secondary diagnosis for outpatient and ED visits.

The secondary objective of this study included gaining a deeper understanding of disease management among influenza episodes. Influenza episodes were defined by the earliest flu diagnosis date (index date) during the season under consideration. To account for the possibility of multiple distinct influenza episodes within one season, a new episode was defined as a flu diagnosis occurring more than 28 days after the index date of the previous episode.

The outcomes of interest for influenza episodes in this study included: the initial point-of-care/setting (index point-of-care) where a patient was first diagnosed with influenza (e.g., outpatient, ED, inpatient, ICU) as well as influenza antiviral prescriptions filled and laboratory tests obtained. Influenza antiviral prescriptions, including Oseltamivir, Amantadine, Zanamivir, Baloxavir, Peramivir, and Rimantadine, were captured using National Drug Codes (NDC) and Healthcare Common Procedure Coding System (HCPCS) codes shown in S1 File. Based on this information, a three-level categorical antiviral variable was created and categorized as ‘No Antiviral Rx’, ‘Within 2 days’ of the index date, and ‘Within 3 to 28 days’ of the index date. Identification of antigen and real-time polymerase chain reaction (RT-PCR) and influenza laboratory tests were identified by using the following CPT codes shown in S1 File. Each of the influenza laboratory tests were categorized as ‘None’, ‘Within 2 days’ of index date, and ‘Within 3 to 28 days’ of the index date.

### Sociodemographic factors

In both databases, sex was captured in the dataset as male and female. In the original Medicaid demographic and eligibility files, race/ethnicity is constructed across 8 categories: White, non-Hispanic; Black, non-Hispanic; Asian, non-Hispanic; American Indian and Alaska Native (AIAN), non-Hispanic; Hawaiian/Pacific Islander; Multiracial, non-Hispanic; Hispanic, all races; Other, non-Hispanic; or Null/missing. For the present analyses, the original 8 racial and ethnic categories were collapsed into the following categories: Non-Hispanic Black, Non-Hispanic White, Hispanic, Asian/AIAN/PI (Asian, non-Hispanic; American Indian and Alaska Native (AIAN), non-Hispanic; Hawaiian/Pacific Islander), Other (Multiracial, non-Hispanic; Other, non-Hispanic), and Missing. Patients from the CDM database were grouped in the same way as in the Medicaid databases. Patient residential regions in the US were categorized as Midwest, Northeast, South, West, and Missing based on patient residential state. Charlson comorbidities [29] were also captured in both databases during the flu season and using a 10-month look-back period from the beginning of each flu season. For example, for a patient in the 2015/2016 flu season, comorbidities were captured from January 1, 2015, to May 31, 2016, in both outpatient and inpatient settings.

### Statistical analyses

Descriptive statistics, including means and standard deviations (SDs) for continuous variables and counts and proportions for categorical variables, were calculated to describe patient characteristics in the Medicaid and CDM databases. Missing demographic data were reported but not imputed, while missing diagnosis information was interpreted as the absence of a condition (e.g., no recorded comorbidity). Patient demographics from both databases were compared using standardized mean differences (SMD). In both databases, the prevalence of influenza and influenza-related healthcare utilization, including outpatient, ED, inpatient, and ICU rates, were calculated per 100,000 patients by season and in the overall study populations. Corresponding 95% confidence intervals (CIs) were calculated using a Poisson distribution. Additionally, outpatient and ED visit rates were reported by sex, race, and US region. Comparisons between both databases were evaluated using Poisson rate ratios (RRs) and their corresponding 95% CIs.

For the analyses focused on influenza episodes, we calculated the proportion of episodes with their index point-of-care in outpatient, ED, inpatient, and ICU settings in the overall study population. Additionally, the index point-of-care for outpatient versus ED settings were calculated by race/ethnicity in both databases. The proportion of influenza episodes with antiviral prescriptions filled and influenza lab tests performed was computed along with the associated binomial exact 95% CI in the overall study population and by sex, race, and region. SMDs were used to compare antiviral prescriptions by demographics between both databases.

Data management and analysis were conducted by using Amazon Redshift Data Warehouse and R Studio Version 4.3.0 for Medicaid data, and on Azure Databricks platform with PySpark and SparkR programming languages for CDM data.

## Results

There were a total of 107,014,615 and 27,623,573 patients between 18–64 years of age from the Medicaid and CDM databases, respectively, that were included in the study (S2 File). Table 1 presents the characteristics of Medicaid and CDM patients, along with their respective SMD values. To facilitate comparability and interpretation between both databases, a negative SMD indicates a lower value in the Medicaid database, while a positive SMD indicates a higher value in the Medicaid database. SMDs close to zero indicate good balance and absolute values of 0.1 start denoting an imbalance between the two populations. Medicaid beneficiaries were, on average, younger (39 vs. 42 years, SMD = -0.21) and had a higher proportion of female patients (60% vs. 50%, SMD = 0.20) compared to the CDM population. The Medicaid population had a higher representation of racial/ethnic minorities compared to the CDM population. Specifically, 19.3% of Medicaid patients identified as non-Hispanic Black (vs. 10.1% in the CDM population, SMD = 0.26), and 15.9% identified as Hispanic (vs. 12.7% in the CDM population, SMD = 0.09). The distribution of US regions was more balanced among Medicaid beneficiaries, while the Southern US region had the highest representation in the CDM population (28% vs. 42%, SMD = -0.28). Although both populations had similar mean Charlson comorbidity scores (0.55 for Medicaid vs. 0.50 for CDM, SMD = 0.04), a slightly higher proportion of Medicaid beneficiaries had at least one comorbidity (26.8% vs. 21.7%, SMD = 0.12). The most prevalent comorbid conditions were chronic obstructive pulmonary disease (COPD) and diabetes without complications in both databases. Regarding the profiles of patients diagnosed with influenza (S3 Table), they displayed similar characteristics to those of the overall population. However, Medicaid had a higher proportion of patients with at least one comorbidity compared to CDM (42% vs. 29%, SMD = 0.24).

**Table 1:** Characteristics of Medicaid and CDM beneficiaries aged 18-64 during the 2015/2016 to 2018/2019 influenza seasons.

	Medicaid		CDM		SMD^1^
	N	%	N	%	
**N**	**107,014,615**		**27,623,573**		
**AGE (MEAN/SD)**	39	14	42	13.2	-0.21
**SEX**					
Female	63,243,958	59.1	13,558,041	49.1	0.2
Male	43,764,751	40.9	14,062,115	50.9	
**RACE**					
Asian	7,069,337	6.6	1,672,185	6.1	0.02
Black	20,639,030	19.3	2,786,992	10.1	0.26
Hispanic	17,036,035	15.9	3,521,659	12.7	0.09
White	44,694,638	41.8	18,415,404	66.7	-0.52
Missing	17,353,315	16.2	1,227,333	4.4	0.4
Other	222,260	0.2	–	–	–
**REGION**					
Midwest	21,649,535	20.2	6,912,037	25	-0.11
Northeast	20,290,127	19	2,559,281	9.3	0.28
South	30,382,719	28.4	11,524,715	41.7	-0.28
West	31,332,928	29.3	5,752,431	20.8	0.2
Missing	3,359,306	3.1	875,109	3.2	-0.01
**CHARLSON INDEX (MEAN/SD)**	0.55	1.34	0.50	1.34	0.04
**CHARLSON COMORBIDITIES**					
**At least one**	28,708,095	26.8	5,997,856	21.7	0.12
AIDS/HIV	910,688	0.9	84,051	0.3	0.08
Cancer	2,491,010	2.3	725,095	2.6	-0.02
Cerebrovascular Disease	2,856,222	2.7	424,470	1.5	0.08
Chronic Pulmonary Disease	14,018,692	13.1	2,226,767	8.1	0.16
Congestive Heart Failure	2,371,530	2.2	343,802	1.2	0.08
Rheumatic Disease	1,344,007	1.3	396,242	1.4	-0.01
Dementia	452,796	0.4	47,741	0.2	0.04
Diabetes with complications	606	0	1,353,995	4.9	-0.32
Diabetes without complications	10,106,230	9.4	2,185,136	7.9	0.05
Metastatic Carcinoma	658,915	0.6	67,996	0.2	0.06
Mild Liver Disease	2,202,916	2.1	623,380	2.3	-0.01
Moderate or Severe Liver Disease	288,289	0.3	92,835	0.3	0
Acute Myocardial Infarction	947,924	0.9	261,519	0.9	0
Paraplegia and Hemiplegia	921,831	0.9	96,335	0.3	0.08
Peptic Ulcer Disease	690,211	0.6	140,764	0.5	0.01
Peripheral Vascular Disease	1,381,108	1.3	310,652	1.1	0.02
Renal Disease	2,554,020	2.4	800,113	2.9	-0.03

^1^Standardized Mean Difference: a negative SMD indicates a lower value in the Medicaid database, while a positive SMD indicates a higher value in the Medicaid database. SMDs close to zero indicate good balance and absolute values of 0.1 start denoting an imbalance between the two populations.

**Fig 1:**
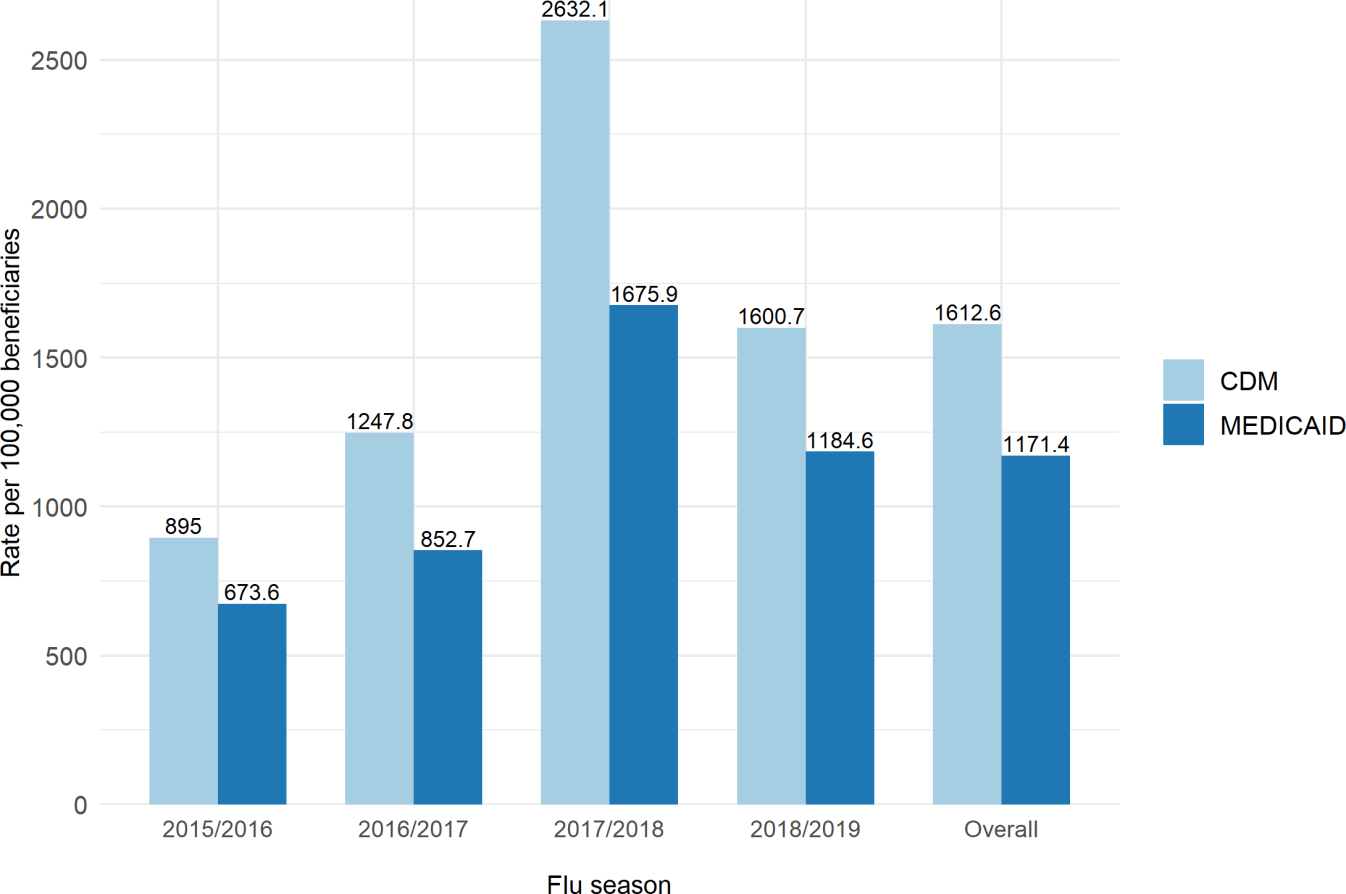
Prevalence of Influenza in Medicaid and CDM beneficiaries aged 18-64 during the study period and 2015/2016 to 2018/2019 influenza seasons.

Fig 1 displays the prevalence of influenza in the overall population and by influenza season. During the entire study period, the prevalence of influenza was 1171.4 per 100,000 in the Medicaid population and 1612.6 per 100,000 in the CDM population. Influenza prevalence increased from the 2015/2016 season to a peak in the 2017/2018 season, followed by a decline in the 2018/2019 season in both databases. Across all seasons, the CDM population consistently had a higher influenza prevalence than the Medicaid population.

**Fig 2:**
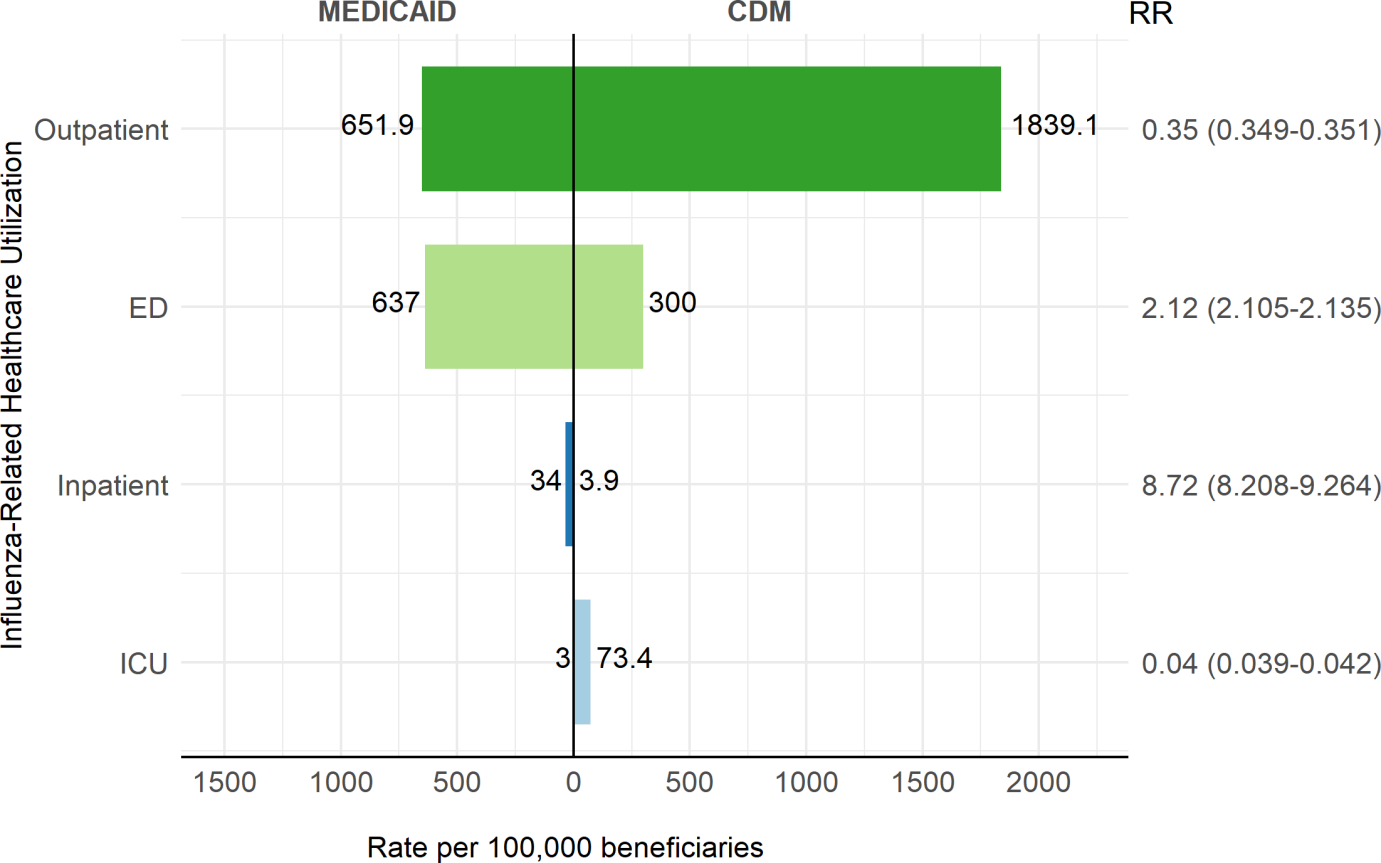
Influenza-related healthcare utilization (Outpatient, ED, Inpatient, and ICU) in Medicaid and CDM beneficiaries aged 18-64, during the 2015/2016 to 2018/2019 influenza seasons.

Fig 2 illustrates influenza-related healthcare utilization (outpatient, ED, inpatient, and ICU) in the Medicaid and CDM populations during the entire study period. For ease of comparison, RR and 95% CI are shown; a RR > 1 indicates a higher value in the Medicaid population, while a RR < 1 indicates a higher value in the CDM population. In the Medicaid population, influenza-related visits in outpatient and ED settings were the most frequent forms of healthcare utilization, with similar rates of 652 and 637 visits per 100,000, respectively. In contrast, the CDM population predominantly utilized outpatient settings, with 1,839 visits per 100,000, which is six times higher than the ED rate of 300 visits per 100,000 in the same population. Notably, the ED rate in the Medicaid population was more than double that of the CDM population (RR: 2.12, 95% CI: 2.11–2.14), whereas the outpatient visit rate was 65% lower (RR: 0.35, 95% CI: 0.349–0.351).

Table 2 describes healthcare utilization across beneficiary characteristics, including sex, race/ethnicity, and US region for both databases. Outpatient influenza rates were higher in the CDM database compared to Medicaid across sex, race/ethnicity, and US region, as indicated by RR < 1. In both databases, females, non-Hispanic Whites, and individuals from the US Southern regions had the highest rates of influenza-related outpatient care. Medicaid beneficiaries had the highest rates of influenza-related ED visits as shown by RR > 1 across all characteristics. Females, non-Hispanic Blacks, and individuals from the US Southern regions had the highest influenza-related ED visit rates in both databases.

**Table 2:** Healthcare utilization rates per 100,000 in Medicaid and CDM beneficiaries aged 18-64 years, stratified by setting (Outpatient, ED, Inpatient, ICU) and grouped by sex, race/ethnicity, and US region during the 2015/2016 to 2018/2019 influenza seasons.

	Medicaid		CDM			
	#	Rate per 100,000 (95% CI)	#	Rate per 100,000 (95% CI)	RR	95% CI
**Outpatient**						
SEX						
Male	196416	448.8 (446.8,450.8)	224151	1594 (1587.4,1600.6)	0.28	0.278 - 0.282
Female	501185	792.5 (790.3,794.7)	287954	2123.9 (2116.1,2131.6)	0.37	0.368 - 0.372
RACE						
White	331905	742.6 (740.1,745.1)	353273	1918.4 (1912,1924.7)	0.39	0.388 - 0.392
Black	86850	420.8 (418,423.6)	50942	1827.8 (1812,1843.7)	0.23	0.227 - 0.233
Hispanic	100993	592.8 (589.2,596.5)	63626	1806.7 (1792.7,1820.7)	0.33	0.327 - 0.333
Asian	47232	668.1 (662.1,674.2)	23679	1416.1 (1398,1434.1)	0.47	0.463 - 0.477
REGION						
Northeast	135696	668.8 (665.2,672.3)	44495	1738.6 (1722.4,1754.7)	0.38	0.376 - 0.384
Midwest	141895	655.4 (652,658.8)	102493	1482.8 (1473.7,1491.9)	0.44	0.436 - 0.444
South	242921	799.5 (796.4,802.7)	293869	2549.9 (2540.7,2559.1)	0.31	0.308 - 0.312
West	163624	522.2 (519.7,524.7)	70355	1223 (1214,1232.1)	0.43	0.426 - 0.434
**ED**						
SEX						
Male	205612	469.8 (467.8,471.8)	36938	262.7 (260,265.4)	1.79	1.77 - 1.81
Female	476065	752.7 (750.6,754.9)	46621	343.9 (340.7,347)	2.19	2.169 - 2.211
RACE						
White	257527	576.2 (574,578.4)	48335	262.5 (260.1,264.8)	2.2	2.179 - 2.221
Black	167729	812.7 (808.8,816.6)	16231	582.4 (573.4,591.3)	1.4	1.378 - 1.423
Hispanic	111193	652.7 (648.9,656.5)	12492	354.7 (348.5,360.9)	1.84	1.806 - 1.874
Asian	28672	405.6 (400.9,410.3)	2399	143.5 (137.7,149.2)	2.83	2.715 - 2.95
REGION						
Northeast	108035	532.5 (529.3,535.6)	5978	233.6 (227.7,239.5)	2.28	2.221 - 2.34
Midwest	148928	687.9 (684.4,691.4)	19220	278.1 (274.1,282)	2.47	2.433 - 2.507
South	246351	810.8 (807.6,814)	46777	405.9 (402.2,409.6)	2	1.98 - 2.02
West	156771	500.3 (497.9,502.8)	11365	197.6 (193.9,201.2)	2.53	2.482 - 2.579
**Inpatient**						
SEX						
Male	14528	33.2 (32.7,33.7)	9188	65.3 (64,66.7)	0.51	0.497 - 0.523
Female	21904	34.6 (34.2,35.1)	11284	83.2 (81.7,84.8)	0.42	0.411 - 0.43
RACE						
White	16111	36 (35.5,36.6)	13850	75.2 (74,76.5)	0.48	0.469 - 0.491
Black	9394	45.5 (44.6,46.4)	3150	113 (109.1,117)	0.4	0.384 - 0.416
Hispanic	4178	24.5 (23.8,25.3)	2060	58.5 (56,61)	0.42	0.398 - 0.443
Asian	1560	22.1 (21,23.2)	615	36.8 (33.9,39.7)	0.6	0.547 - 0.659
REGION						
Northeast	8641	42.6 (41.7,43.5)	2363	92.3 (88.6,96.1)	0.46	0.44 - 0.481
Midwest	9486	43.8 (42.9,44.7)	5474	79.2 (77.1,81.3)	0.55	0.532 - 0.569
South	10451	34.4 (33.7,35.1)	9188	79.7 (78.1,81.4)	0.43	0.418 - 0.442
West	6995	22.3 (21.8,22.8)	3395	59 (57,61)	0.38	0.365 - 0.396
**ICU**						
SEX						
Male	1400	3.2 (3,3.4)	489	3.5 (3.2,3.8)	0.91	0.821 - 1.009
Female	1825	2.9 (2.8,3)	602	4.4 (4.1,4.8)	0.66	0.602 - 0.724
RACE						
White	1633	3.7 (3.5,3.8)	697	3.8 (3.5,4.1)	0.97	0.888 - 1.06
Black	655	3.2 (2.9,3.4)	179	6.4 (5.5,7.4)	0.5	0.424 - 0.59
Hispanic	347	2 (1.8,2.3)	134	3.8 (3.2,4.4)	0.53	0.434 - 0.647
Asian	122	1.7 (1.4,2)	24	1.4 (0.9,2)	1.21	0.781 - 1.874
REGION						
Northeast	552	2.7 (2.5,3)	69	2.7 (2.1,3.3)	1	0.779 - 1.284
Midwest	1077	5 (4.7,5.3)	254	3.7 (3.2,4.1)	1.35	1.177 - 1.548
South	862	2.8 (2.6,3)	583	5.1 (4.6,5.5)	0.55	0.495 - 0.611
West	667	2.1 (2,2.3)	181	3.1 (2.7,3.6)	0.68	0.577 - 0.801

**Fig 3:**
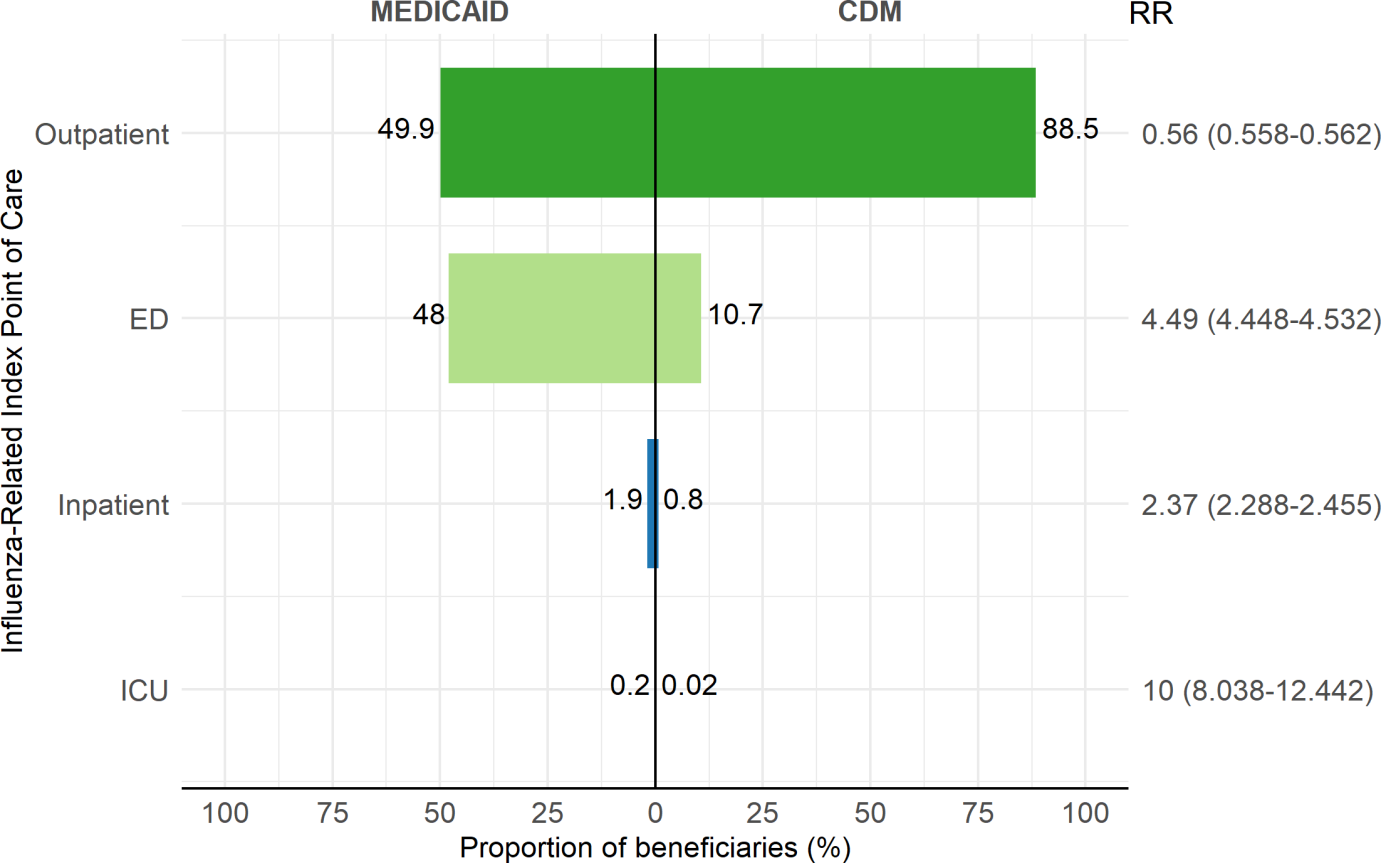
Influenza index point-of-care healthcare settings in Medicaid and CDM beneficiaries aged 18-64, during the 2015/2016 to 2018/2019 influenza seasons.

Fig 3 shows the distribution of index point-of-care for influenza episodes (patients with an influenza diagnosis) across the various healthcare settings. Among Medicaid beneficiaries, the influenza index point-of-care was nearly evenly split between outpatient (49.9%) and ED (48.0%) settings. In contrast, in the CDM database, the index point-of-care for influenza was predominantly in the outpatient setting (88.5%), which was eight times higher than in the ED (10.7%).

**Fig 4:**
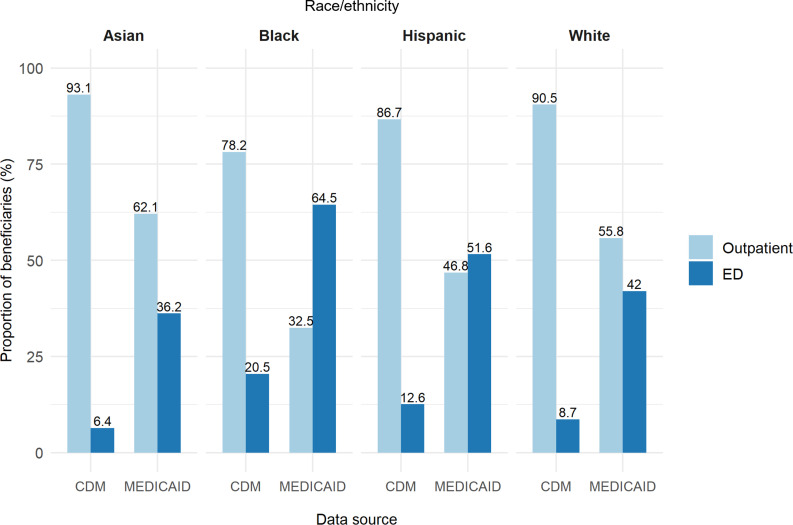
Influenza index point-of-care healthcare settings by race/ethnicity in Medicaid and CDM influenza episodes aged 18-64, during the 2015/2016 to 2018/2019 influenza seasons.

Fig 4 shows the proportion of influenza index point-of-care visits in outpatient versus ED settings by race/ethnicity throughout the study period*.* In the Medicaid population, Black (64.5%) and Hispanic (51.6%) patients predominantly used the ED as their index point-of-care for influenza, while White (55.8%) and Asian (62.1%) beneficiaries predominantly used outpatient settings. In the CDM population, the outpatient setting was the predominant index point- of-care across all racial groups, ranging from 78.2% for Black patients and 93.1% for Asian patients. Influenza index point-of-care by sex, race/ethnicity, and US region for all healthcare settings (e.g., inpatient, ICU) are shown in S4 Table.

**Fig 5:**
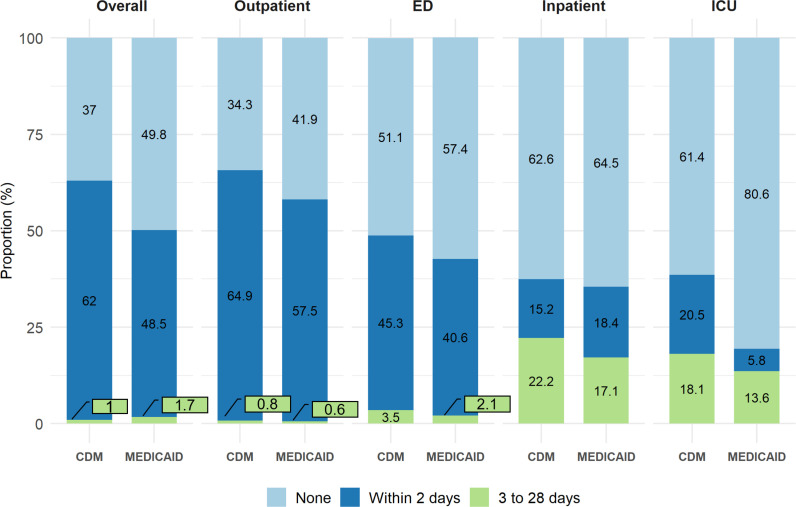
Influenza antiviral prescription patterns in the overall study population and across all index point-of-care healthcare settings in Medicaid and CDM influenza episodes aged 18-64, during the 2015/2016 to 2018/2019 influenza seasons.

Fig 5 shows the distribution of influenza antiviral prescriptions filled in the overall population and across the various influenza index point-of-care settings. Overall, a greater proportion of Medicaid beneficiaries (49.8%) did not fill any influenza antiviral prescription compared to the CDM population (37%). This pattern was consistent across all influenza index point-of-care settings. However, among those with an inpatient index point-of-care, the proportion who filled an influenza antiviral prescription was comparable between Medicaid (35.5%) and CDM (37.4%). In both populations, the highest percentage of influenza antiviral prescriptions were filled by patients whose index point-of-care was an outpatient setting. Among patients with outpatient and ED as their influenza index point-of-care, the majority of influenza antiviral prescriptions were filled within 2 days. In contrast, for patients whose influenza index point-of-care was inpatient or ICU, influenza antiviral prescriptions were typically filled later, within 3–28 days. Table 3 displays the proportion of influenza antiviral prescriptions by sex, race/ethnicity, and gender. Across all demographic groups, a higher proportion of CDM patients filled an influenza antiviral prescription within 2 days of the index date compared to Medicaid patients. Among Black beneficiaries, 54.3% in Medicaid and 40.3% in CDM did not fill a flu antiviral prescription, which was the highest proportion among all racial and ethnic groups in both databases.

**Table 3:** Proportion of antiviral prescription by sex, race/ethnicity, and US region in Medicaid and CDM influenza episodes aged 18-64, during the 2015/2016 to 2018/2019 influenza seasons.

	Medicaid		CDM		
	#	% (95% CI)	#	% (95% CI)	SMD
**No Antiviral Rx**					
SEX					
Female	441892	49.4 (49.3, 49.5)	94987	38.2 (38, 38.4)	0.23
Male	189728	50.9 (50.7, 51.1)	71004	36.1 (35.8, 36.4)	0.3
RACE					
Asian	32299	46.3 (45.9, 46.6)	7532	36.8 (36, 37.6)	0.19
Black	127828	54.3 (54.1, 54.5)	17901	40.3 (39.7, 40.9)	0.28
Hispanic	92258	47.4 (47.2, 47.6)	19629	35.3 (34.8, 35.8)	0.25
White	269106	49.8 (49.7, 49.9)	112252	36.6 (36.4, 36.8)	0.27
REGION					
Midwest	133370	50.9 (50.7, 51.1)	38953	44.4 (43.9, 44.9)	0.13
Northeast	106218	47.1 (46.8, 47.3)	14712	38.8 (38.2, 39.4)	0.17
South	233821	51.4 (51.2, 51.5)	83460	32.3 (32.1, 32.5)	0.39
West	141327	48.4 (48.2, 48.6)	25754	42.3 (41.8, 42.8)	0.12
**Within 2 days**					
SEX					
Female	438797	49 (48.9, 49.1)	150117	60.4 (60.2, 60.5)	-0.23
Male	176105	47.2 (47.1, 47.4)	123537	62.8 (62.6, 63)	-0.32
RACE					
Asian	36429	52.2 (51.8, 52.5)	12723	62.2 (61.6, 62.7)	-0.2
Black	102385	43.5 (43.3, 43.7)	25716	57.9 (57.5, 58.2)	-0.29
Hispanic	98999	50.9 (50.7, 51.1)	35280	63.4 (63.1, 63.7)	-0.25
White	263256	48.7 (48.6, 48.8)	191114	62.2 (62.1, 62.4)	-0.27
REGION					
Midwest	123634	47.2 (47, 47.4)	48003	54.7 (54.5, 54.9)	-0.15
Northeast	115520	51.2 (51, 51.4)	22703	59.9 (59.5, 60.3)	-0.18
South	215214	47.3 (47.1, 47.4)	171213	66.3 (66.2, 66.5)	-0.39
West	145225	49.7 (49.6, 49.9)	34426	56.5 (56.2, 56.9)	-0.14
**Within 3–28 days**					
SEX					
Female	14165	1.6 (1.6, 1.6)	3595	1.4 (1.4, 1.5)	0.02
Male	6933	1.9 (1.8, 1.9)	2157	1.1 (1, 1.1)	0.07
RACE					
Asian	1099	1.6 (1.5, 1.7)	208	1 (1, 1.1)	0.05
Black	5175	2.2 (2.1, 2.3)	832	1.9 (1.8, 1.9)	0.02
Hispanic	3344	1.7 (1.7, 1.8)	709	1.3 (1.2, 1.3)	0.03
White	8091	1.5 (1.5, 1.5)	3682	1.2 (1.1, 1.3)	0.03
REGION					
Midwest	4970	1.9 (1.8, 2)	856	1 (0.9, 1)	0.08
Northeast	3990	1.8 (1.7, 1.8)	463	1.2 (1.2, 1.3)	0.05
South	6265	1.4 (1.3, 1.4)	3489	1.4 (1.3, 1.4)	0
West	5376	1.8 (1.8, 1.9)	698	1.1 (1.1, 1.2)	0.06

**Fig 6:**
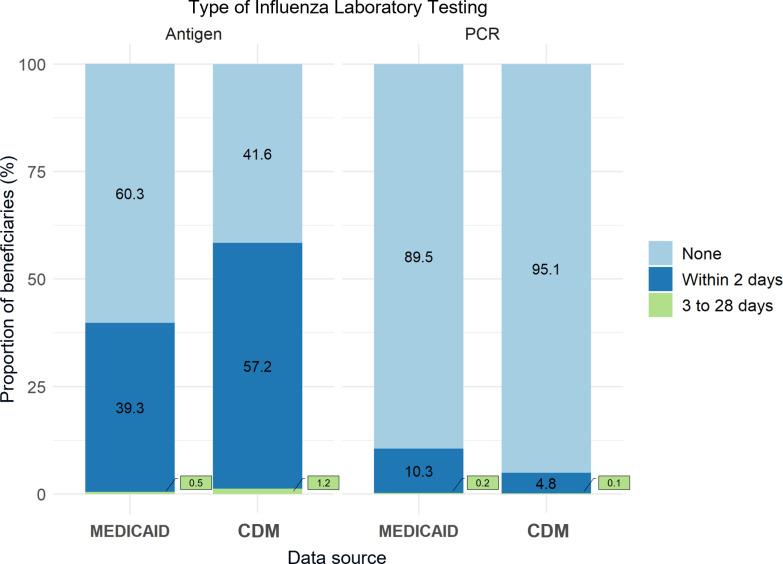
Influenza laboratory testing performed in Medicaid and CDM influenza episodes aged 18-64, during the 2015/2016 to 2018/2019 influenza seasons.

Fig 6 shows the proportion of influenza laboratory testing performed across influenza episodes in Medicaid and CDM populations during the study period. Influenza antigen testing was the most common laboratory test performed in Medicaid and CDM populations. However, Medicaid beneficiaries had fewer antigen tests completed than the CDM population (40% vs. 58%, respectively). The characteristics of influenza antigen testing and RT-PCR testing by sex, race/ethnicity, US region, and index point-of-care are presented in S5 and S6 Tables, respectively.

## Discussion

This comparative analysis between Medicaid and CDM patients revealed underlying disparities in influenza healthcare utilization and treatment between and within both cohorts. Medicaid beneficiaries had a higher representation of racial/ethnic minorities, lower rates of influenza-related outpatient care, higher rates of influenza-related ED care, and a lower proportion of influenza episodes with an antiviral prescription filled compared to the CDM population. Moreover, Medicaid beneficiaries sought the ED as their index point-of-care for influenza at a much higher frequency than the CDM population. The prevalence of influenza in this study was 1.17% and 1.61% in the Medicaid and CDM populations, respectively. Our study’s prevalence is much lower than the CDC’s reported median incidence of 8.8% for adults aged 18–64 [30]. The CDC’s estimates are based on data from the Influenza Hospitalization Surveillance Network (FluSurv-NET), which captures laboratory-confirmed cases and is supplemented with mathematical models to account for unreported cases in individuals who do not seek care or are not formally tested [31]. In contrast, our claims-based analysis only captures influenza cases for individuals who sought medical care and were assigned an official influenza diagnostic code. This methodological difference likely explains the discrepancy in influenza prevalence between our findings and the CDC’s estimates.

Although our study identified a lower overall prevalence of influenza compared to what is commonly reported in the literature, we did observe a higher prevalence and greater influenza-related ED utilization in the Southern US, consistent with previous studies. The underlying reasons for the greater flu and ED burden in the South are not fully understood. However, the South is the most populated region in the US, with two of the top three most populous states (Florida and Texas) [32]. The southern region of the US is home to several large metropolitan areas with high population density, including cities such as Atlanta, Miami, and Houston. These areas are also popular domestic and international travel destinations, which can lead to a more rapid spread of infectious diseases due to increased person-to-person contact [33]. Additionally, the South has a higher retirement-age population, which is more at risk for severe cases of influenza [34,35]. Furthermore, lower influenza vaccination rates in the Southern states may contribute to the higher flu burden observed in this region [36]. Combined with these factors, higher ED utilization in the Southern US may be driven by social determinants of health, such as poverty, limited healthcare provider availability, and a greater proportion of rural communities where access to primary care is restricted [37].

Although there were differences in influenza-related outpatient and ED rates between the Medicaid and CDM populations, both databases revealed that non-Hispanic Whites had the highest rates of influenza-related outpatient care. Additionally, non-Hispanic Blacks and Hispanics exhibited the highest rates of influenza-related ED visits in both cohorts. The patterns of influenza-related healthcare utilization observed in this study align with the well-documented disparities in access to care across insurance groups and racial/ethnic minorities. In their recent exploration of racial/ethnic disparities in ED utilization, Parast et al. found that Black and Hispanic patients, compared to their White counterparts, were more likely to report visiting the ED for an ongoing health condition, as well as report not having a usual source of care/primary care doctor [38]. Furthermore, research has demonstrated that Black patients are less likely than White patients to utilize primary care as their customary source of healthcare, a disparity linked to differences in socioeconomic status, access to primary care services, and medical mistrust [39]. Moreover, higher ED use among Black patients may also be influenced by clinician bias which can reduce engagement with primary care services and contribute to increased reliance on EDs [40]. Despite national efforts to mitigate the gap in access to care across racial and ethnic groups, recent research shows that racial/ethnic disparities in timely medical care unrelated to cost have worsened in the last 20 years. Factors including lack of transportation and long waiting times have been highlighted as significant barriers [41]. Such patterns underscore the need for targeted interventions to improve access to primary care to ensure equitable healthcare across all population demographics.

This study revealed disparities in influenza-related ED visits between Medicaid and CDM populations, highlighting the complexities and factors influencing patients’ care-seeking behavior. A recent qualitative study on frequent ED users among Medicaid recipients reported that limited access to primary care providers (PCP), negative personal experiences with the healthcare system, low socioeconomic status, and significant chronic mental and physical disease burdens were key reasons for their consistent ED visits [42]. Moreover, some Medicaid beneficiaries in that study also voiced concerns about differential treatment by PCP staff due to their “public insurance” status [13]. These multifactorial disparities in access to care warrant further investigation to ensure that all patients can access preventive healthcare and its associated benefits.

To address disparities in healthcare and improve primary care access, interventions such as transportation support have been shown to reduce missed medical appointments. [43]. While Medicaid offers Non-Emergency Medical Transportation (NEMT) services that include public transportation, personal vehicle reimbursement, and ride services, awareness of this benefit among beneficiaries appears to be low [44]. The Centers for Medicare & Medicaid Services (CMS) has encouraged states to collaborate with health plans and providers to increase awareness of NEMT services [45]. However, challenges remain for beneficiaries who utilize NEMT, such as state-level differences in policies, as well as issues with timeliness and reliability of transportation [46]. Enhancing the flexibility and reliability of NEMT services could improve healthcare access for Medicaid beneficiaries. In addition to assisting with transportation support, expanding community health worker (CHW) programs can also enhance primary care access [47]. CHWs are frontline healthcare workers who are either trusted community members or have a deep understanding of the populations they serve, which helps foster trust and strengthen patient-provider relationships [48]. Furthermore, cultural competence interventions can improve communication, trust, and patient satisfaction, helping to reduce racial and ethnic health disparities [49]. Additionally, diversifying the healthcare workforce further strengthens patient-provider relationships, as patients often report better care experiences with providers who share similar cultural backgrounds [49].

In this study, 49.8% of Medicaid patients and 37.0% of CDM patients did not fill an antiviral prescription during their influenza episode. Data from the Behavioral Risk Factor Surveillance System (BRFSS) between January and April 2011 found that only 34% of individuals with a self-reported physician diagnosis of influenza received an antiviral [50]. Similarly, a study focusing on high-risk outpatients enrolled in the US Influenza Vaccine Effectiveness Network from 2011 to 2016 found that 37% of RT-PCR-confirmed influenza cases were prescribed antiviral medications [51]. Another study of physician offices and hospital outpatient settings for both children and adults between 2009 and 2016 identified that, overall, 39.4% of influenza patients received antivirals, with rates varying from 43.3% in the 2013–2014 season to 52.7% in the 2014–2015 season [52]. More recently, a study using MarketScan data from the 2014–2016 influenza seasons found that 60.2% of patients diagnosed with influenza received antiviral treatment [53], a percentage comparable to that observed for influenza episodes with an influenza antiviral prescription filled in the CDM cohort of this study. Our study focuses on more contemporary data and includes insured populations (whether private or public) compared to the general population. Notably, the majority of our data comes from the 2017–2018 and 2018–2019 flu seasons during which Oseltamivir prescriptions experienced a notable increase compared to prior years [54]. Despite these improvements, evidence suggests that influenza antivirals may be underutilized, especially in high-risk patients [55]. While reasons behind influenza antiviral underutilization may be multifactorial, ensuring timely antiviral administration is critical for reducing adverse influenza outcomes. Addressing this gap could lead to improved patient outcomes and more efficient use of healthcare resources. Although there is no specific literature on influenza testing disparities between Medicaid and commercial populations, evidence shows that commercial insurance enrollees receive more low-value care, partly due to differences in reimbursement incentives [56]. Lower influenza testing rates in the Medicaid population may also stem from limited access to primary care and point-of-care diagnostic tools and laboratory services, which are crucial for timely care but may be less available in resource-limited healthcare settings [57].

The results of this study should be interpreted considering some limitations. First, there is the possibility of misclassifying influenza cases due to inaccuracies in recording diagnostic codes. Second, we defined influenza cases strictly as those with a confirmed flu diagnosis, excluding patients who presented with influenza-like symptoms but did not receive a specific flu diagnosis. Third, the lack of lab result information may have led to the misdiagnosis of flu cases and the exclusion of patients who tested positive for influenza but were not identified with a corresponding diagnosis code in the data [58]. Additionally, by employing a stringent definition for inpatient flu cases based solely on the primary diagnosis code, there is a potential for undercounting inpatient flu cases. Another limitation of this study is the absence of adjustments for socioeconomic status. While the Medicaid database primarily represents lower-income individuals due to its income-based eligibility, the CDM database includes a commercially insured population spanning a broader range of income levels. This difference may partially explain some of the observed disparities in influenza-related outpatient versus ED utilization. Furthermore, our data only indicates whether a patient filled an antiviral prescription, and it does not confirm the actual medication consumption by beneficiaries. Also, baseline influenza vaccination rates were not measured in this study, which could influence the prevalence of influenza and the use of antiviral medications in the study populations. Prior studies have shown that vaccination rates differ across insurance types, with approximately 33% of Medicaid beneficiaries and 41% of privately insured adults receiving the influenza vaccine, compared to 16% of uninsured adults [16]. These disparities may partially explain variations in healthcare outcomes and antiviral use. Moreover, state-specific Medicaid policies were not considered in these analyses and may have influenced the healthcare utilization patterns observed. Additionally, during the 2015/2016 flu season, the Medicaid dataset had a smaller sample size because some states had not fully transitioned to the TAF RIF system [59]. However, most of the analyses focus on flu seasons after 2015/2016, when data completeness improved, so this limitation is unlikely to significantly affect the findings. Although our findings provide insights into influenza-related healthcare utilization, they may not be generalizable to populations without insurance or those with inconsistent insurance coverage. Additionally, the outcomes in our study reflect billed healthcare encounters and do not capture unbilled care or patient-reported experiences which hinders our ability to assess other aspects of healthcare access and quality of care.

Despite several limitations inherent to the dataset and study design, this study has many strengths. Medicaid and CDM claims offer an extensive demographic and clinical information repository, including age, sex, race/ethnicity, various medical services rendered, and prescription histories. Since claims data are collected from providers, the likelihood of self-reporting biases is low, ensuring a more accurate representation of healthcare encounters. Moreover, the substantial sample size of our study and the longitudinal nature of the data provide robustness and granularity that is often lacking in smaller cohort or cross-sectional studies. Many existing studies on influenza outcomes rely on population-based surveys (e.g., BRFSS, FNY), CDC data, or employ an all-payer approach, which may be limited by recall bias or the inability to track individual patient care over time. In contrast, our focused methodology allows for the identification of detailed healthcare utilization patterns and differences in influenza care across Medicaid and commercial insurance populations, addressing an important gap in the literature. Both the Medicaid and CDM datasets include data from all 50 states, ensuring broad geographic representation. While these datasets are not specifically designed to track influenza activity or patterns, they are valuable for examining healthcare utilization and outcomes across diverse populations. The nationwide scope of these datasets allows for meaningful analyses of influenza-related healthcare utilization that can complement existing surveillance systems focused on influenza activity.

## Conclusion

The results of this study reveal disparities in influenza-related healthcare utilization between Medicaid and CDM populations, notably regarding differences in outpatient and ED care. Medicaid beneficiaries had higher rates of influenza-related ED care and lower rates of influenza-related outpatient care compared to the CDM population. Moreover, a higher proportion of Medicaid influenza episodes did not fill an influenza antiviral prescription compared to the CDM population, though both populations underutilized influenza antivirals. Addressing these disparities in influenza-related care is essential to achieving equitable healthcare for all patients, regardless of their insurance status or demographic background.

## Supporting information

S1 File Data specifications and coding details for study outcomes. (XLSX)

S2 File Inclusion flow chart. (DOCX)

S3 Table Characteristics of Medicaid and CDM beneficiaries with influenza aged 18–64 during the 2015/2016–2018/2019 influenza seasons. (DOCX)

S4 Table Influenza index point-of-care by sex, race/ethnicity, US region in Medicaid and CDM beneficiaries with influenza aged 18–64, during the 2015/2016–2018/2019 influenza seasons. (DOCX)

S5 Table Influenza antigen testing by sex, race/ethnicity, US region, and index setting. (DOCX)

S6 Table Proportion of influenza RT-PCR by sex, race/ethnicity, US Region, and index setting. (DOCX)

S7 File Data values for tables and figures. (XLSX)
