# Supplementary figures and images for: Comparative analysis of influenza healthcare disparities in the United States using retrospective administrative claims from Medicaid and commercial databases, 2015–2019
Source: PLoS One. 2025 May 22;20(5):e0321208. doi: 10.1371/journal.pone.0321208 (PMC12097570; doi:10.1371/journal.pone.0321208)

S2 File: Attrition figure, Medicaid and CDM databases


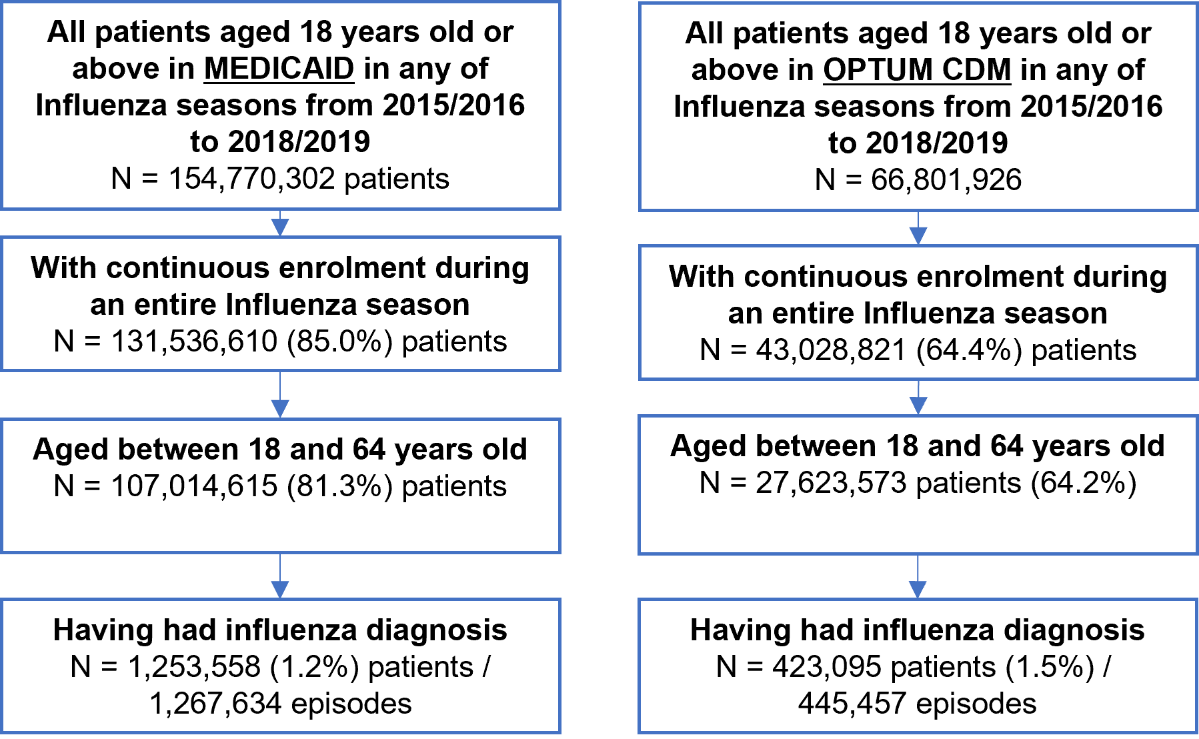

Supplement: S2 File — (DOCX) [file pone.0321208.s002.docx]
