# Supplementary material for: Comparative analysis of influenza healthcare disparities in the United States using retrospective administrative claims from Medicaid and commercial databases, 2015–2019
Source: PLoS One. 2025 May 22;20(5):e0321208. doi: 10.1371/journal.pone.0321208 (PMC12097570; doi:10.1371/journal.pone.0321208)
Supplement: S3 Table — (DOCX) [file pone.0321208.s003.docx]

S3 Table: Characteristics of Medicaid and CDM Beneficiaries With Influenza Aged 18-64 During the 2015/2016 to 2018/2019 Influenza Seasons

|  | **Medicaid** | | **CDM** | | **SMD** |
| --- | --- | --- | --- | --- | --- |
|  | **N** | **%** | **N** | **%** |  |
| **N** | **1,253,558** |  | **445,457** |  |  |
| **AGE (MEAN/SD)** | 37 | 13 | 40.9 | 12.9 | 0.3 |
| **SEX** |  |  |  |  |  |
| Female | 884463 | 71 | 248699 | 55.8 | -0.32 |
| Male | 369081 | 29 | 196698 | 44.2 |  |
| **RACE** |  |  |  |  |  |
| Asian | 68765 | 5.5 | 20463 | 4.6 | 0.04 |
| Black | 233007 | 19 | 44449 | 10 | 0.26 |
| Hispanic | 192440 | 15 | 55618 | 12.5 | 0.07 |
| White | 534866 | 43 | 307048 | 68.9 | -0.54 |
| Missing | 221636 | 18 | 17879 | 4 | 0.46 |
| Other | 2844 | 0.2 | - | - | - |
| **REGION** |  |  |  |  |  |
| Midwest | 259600 | 21 | 87812 | 19.7 | 0.03 |
| Northeast | 223249 | 18 | 37878 | 8.5 | 0.28 |
| South | 449482 | 36 | 258162 | 58 | -0.45 |
| West | 288871 | 23 | 60878 | 13.7 | 0.24 |
| Missing | 32356 | 2.6 | 727 | 0.2 | 0.21 |
| **CHARLSON INDEX (MEAN/SD)** | 0.76 | 1.44 | 0.7 | 1.5 | 0.09 |
| **CHARLSON COMORBIDITIES** |  |  |  |  |  |
| **At least one** | 502057 | 40.1 | 127,665 | 28.7 | 0.24 |
| AIDS/HIV | 13805 | 1.1 | 1638 | 0.4 | -0.08 |
| Cancer | 32159 | 2.6 | 13410 | 3 | 0.02 |
| Cerebrovascular Disease | 38434 | 3.1 | 8090 | 1.8 | -0.08 |
| Chronic Pulmonary Disease | 330457 | 26 | 59947 | 13.5 | -0.32 |
| Congestive Heart Failure | 38806 | 3.1 | 8016 | 1.8 | -0.08 |
| Rheumatic Disease | 27156 | 2.2 | 9662 | 2.2 | 0 |
| Dementia | 4235 | 0.3 | 668 | 0.1 | -0.04 |
| Diabetes with complications | ---^a^ | <0.1% | 26742 | 6 | - |
| Diabetes without complications | 155985 | 12 | 41546 | 9.3 | -0.09 |
| Metastatic Carcinoma | 6146 | 0.5 | 1178 | 0.3 | -0.03 |
| Mild Liver Disease | 27280 | 2.2 | 14612 | 3.3 | 0.07 |
| Moderate or Severe Liver Disease | 2893 | 0.2 | 1795 | 0.4 | 0.04 |
| Acute Myocardial Infarction | 14346 | 1.1 | 5620 | 1.3 | 0.02 |
| Paraplegia and Hemiplegia | 9726 | 0.8 | 1491 | 0.3 | -0.07 |
| Peptic Ulcer Disease | 12352 | 1 | 3372 | 0.8 | -0.02 |
| Peripheral Vascular Disease | 18314 | 1.5 | 5601 | 1.3 | -0.02 |
| Renal Disease | 36890 | 2.9 | 16945 | 3.8 | 0.05 |

^1^Standard Mean Difference: a negative SMD indicates a lower value in the Medicaid database, while a positive SMD indicates a higher value in the Medicaid database
