# Supplementary material for: Comparative analysis of influenza healthcare disparities in the United States using retrospective administrative claims from Medicaid and commercial databases, 2015–2019
Source: PLoS One. 2025 May 22;20(5):e0321208. doi: 10.1371/journal.pone.0321208 (PMC12097570; doi:10.1371/journal.pone.0321208)
Supplement: S4 Table — (DOCX) [file pone.0321208.s004.docx]

S4 Table: Influenza Index Point of Care By Sex, Race/Ethnicity, US Region in Medicaid and CDM Beneficiaries With Influenza Aged 18-64, During the 2015/2016 to 2018/2019 Influenza Seasons

|  |  |  | 18-64 years | | | | |
| --- | --- | --- | --- | --- | --- | --- | --- |
|  | Medicaid | | CDM | |  | | |
|  | **#** | **Proportion** | **#** | **Proportion** | **SMD** | **RR** | **CI** |
| Outpatient | | | | | | | |
| SEX |  |  |  |  |  | |  |
| Female | 453833 | 50.7 (50.6, 50.8) | 220876 | 88.8 (88.4,89.2) | -0.91 | 0.57 | 0.567 - 0.573 |
| Male | 178320 | 47.8 (47.7, 48) | 174385 | 88.7 (88.2,89.1) | -0.98 | 0.54 | 0.536 - 0.544 |
| RACE |  |  |  |  |  |  | |
| Asian | 43390 | 62.1 (61.8, 62.5) | 19054 | 93.1 (91.8,94.4) | -0.8 | 0.67 | 0.659 - 0.682 |
| Black | 76583 | 32.5 (32.3, 32.7) | 34754 | 78.2 (77.4,79) | -1.04 | 0.42 | 0.415 - 0.425 |
| Hispanic | 90997 | 46.8 (46.5, 47) | 48246 | 86.7 (86,87.5) | -0.93 | 0.54 | 0.534 - 0.546 |
| White | 301661 | 55.8 (55.7, 55.9) | 277802 | 90.5 (90.1,90.8) | -0.85 | 0.62 | 0.617 - 0.623 |
| Missing | 118266 | 52.7 (52.5, 52.9) | 15461 | 86.5 (85.1,87.8) | -0.79 | 0.61 | 0.6 - 0.62 |
| Other | 1265 | 44 (42.2, 45.9) |  |  | 1.25 |  | |
| REGION |  |  |  |  |  |  | |
| Midwest | 124282 | 47.4 (47.2, 47.6) | 76208 | 86.8 (86.2,87.4) | -0.92 | 0.55 | 0.545 - 0.555 |
| Northeast | 123307 | 54.6 (54.4, 54.8) | 34135 | 90.1 (89.2,91.1) | -0.86 | 0.61 | 0.603 - 0.617 |
| South | 224781 | 49.4 (49.2, 49.5) | 230607 | 89.3 (89,89.7) | -0.96 | 0.55 | 0.547 - 0.553 |
| West | 147755 | 50.6 (50.4, 50.8) | 53766 | 88.3 (87.6,89.1) | -0.9 | 0.57 | 0.564 - 0.576 |
| Missing | 12037 | 36.8 (36.3, 37.3) | 601 | 82.7 (76.1,89.3) | -1.06 | 0.44 | 0.405 - 0.478 |
| ED | | | | | | | |
| SEX |  |  |  |  |  | |  |
| Female | 424784 | 47.5 (47.4, 47.6) | 25877 | 10.4 (10.3,10.5) | 0.9 | 4.57 | 4.513 - 4.628 |
| Male | 183402 | 49.2 (49, 49.4) | 20622 | 10.5 (10.3,10.6) | 0.93 | 4.69 | 4.623 - 4.758 |
| RACE |  |  |  |  |  |  | |
| Asian | 25243 | 36.2 (35.8, 36.5) | 1309 | 6.4 (6.1,6.7) | 0.78 | 5.66 | 5.354 - 5.983 |
| Black | 151714 | 64.5 (64.3, 64.6) | 9097 | 20.5 (20,20.9) | 0.99 | 3.15 | 3.084 - 3.217 |
| Hispanic | 100410 | 51.6 (51.4, 51.8) | 6982 | 12.6 (12.3,12.8) | 0.92 | 4.1 | 4.002 - 4.201 |
| White | 226829 | 42 (41.8, 42.1) | 26852 | 8.7 (8.6,8.8) | 0.83 | 4.83 | 4.769 - 4.891 |
| Missing | 102431 | 45.6 (45.4, 45.8) | 2263 | 12.7 (12.1,13.2) | 0.78 | 3.59 | 3.444 - 3.743 |
| Other | 1564 | 54.5 (52.6, 56.3) |  |  | 1.55 |  | |
| REGION |  |  |  |  |  |  | |
| Midwest | 130794 | 49.9 (49.7, 50.1) | 10699 | 12.2 (12,12.4) | 0.89 | 4.09 | 4.01 - 4.171 |
| Northeast | 95789 | 42.4 (42.2, 42.6) | 3353 | 8.9 (8.6,9.2) | 0.83 | 4.76 | 4.599 - 4.927 |
| South | 222765 | 48.9 (48.8, 49.1) | 25861 | 10 (9.9,10.1) | 0.94 | 4.89 | 4.827 - 4.953 |
| West | 138867 | 47.6 (47.4, 47.8) | 6477 | 10.6 (10.4,10.9) | 0.89 | 4.49 | 4.38 - 4.603 |
| Missing | 19976 | 61.1 (60.6, 61.6) | 113 | 15.5 (12.7,18.4) | 1.06 | 3.94 | 3.275 - 4.74 |
| Inpatient | | | | | | | |
| SEX |  |  |  |  |  | |  |
| Female | 14749 | 1.6 (1.6, 1.7) | 1904 | 0.8 (0.7,0.8) | 0.07 | 2 | 1.907 - 2.098 |
| Male | 9862 | 2.6 (2.6, 2.7) | 1652 | 0.8 (0.8,0.9) | 0.14 | 3.25 | 3.085 - 3.424 |
| RACE |  |  |  |  |  |  | |
| Asian | 1098 | 1.6 (1.5, 1.7) | 97 | 0.5 (0.4,0.6) | 0.11 | 3.2 | 2.6 - 3.938 |
| Black | 6525 | 2.8 (2.7, 2.8) | 584 | 1.3 (1.2,1.4) | 0.11 | 2.15 | 1.975 - 2.34 |
| Hispanic | 2901 | 1.5 (1.4, 1.5) | 381 | 0.7 (0.6,0.8) | 0.08 | 2.14 | 1.923 - 2.381 |
| White | 10618 | 2 (1.9, 2) | 2344 | 0.8 (0.7,0.8) | 0.1 | 2.5 | 2.391 - 2.614 |
| Missing | 3433 | 1.5 (1.5, 1.6) | 150 | 0.8 (0.7,1) | 0.07 | 1.88 | 1.596 - 2.214 |
| Other | 36 | 1.3 (0.9, 1.7) |  |  | 0.16 |  | |
| REGION |  |  |  |  |  |  | |
| Midwest | 6026 | 2.3 (2.2, 2.4) | 879 | 1 (0.9,1.1) | 0.1 | 2.3 | 2.143 - 2.469 |
| Northeast | 6150 | 2.7 (2.7, 2.8) | 387 | 1 (0.9,1.1) | 0.13 | 2.7 | 2.436 - 2.992 |
| South | 7038 | 1.5 (1.5, 1.6) | 1649 | 0.6 (0.6,0.7) | 0.09 | 2.5 | 2.369 - 2.638 |
| West | 4762 | 1.6 (1.6, 1.7) | 628 | 1 (1,1.1) | 0.05 | 1.6 | 1.472 - 1.739 |
| Missing | 635 | 1.9 (1.8, 2.1) | 13 | 1.8 (0.8,2.8) | 0.01 | 1.06 | 0.612 - 1.836 |
| ICU | | | | | | | |
| SEX |  |  |  |  |  | |  |
| Female | 1488 | 0.2 (0.2, 0.2) | 42 | 0 (0,0) | 0.06 | 0 | |
| Male | 1182 | 0.3 (0.3, 0.3) | 39 | 0 (0,0) | 0.08 | 0 | 0 - 0 |
| RACE |  |  |  |  |  |  | |
| Asian | 96 | 0.1 (0.1, 0.2) | ---^a^ | ---^a^ | ---^a^ | ---^a^ | ---^a^ |
| Black | 566 | 0.2 (0.2, 0.3) | 14 | 0 (0,0) | 0.06 | 0 | 0 - 0 |
| Hispanic | 293 | 0.2 (0.1, 0.2) | 9 | 0 (0,0) | 0.06 | 0 | 0 - 0 |
| White | 1345 | 0.2 (0.2, 0.3) | 50 | 0 (0,0) | 0.06 | 0 | 0 - 0 |
| Missing | 363 | 0.2 (0.1, 0.2) | 5 | 0 (0,0.1) | 0.06 | 0 | 0 - 0 |
| Other |  |  |  |  |  |  | |
| REGION |  |  |  |  |  |  | |
| Midwest | 872 | 0.3 (0.3, 0.4) | 26 | 0 (0,0) | 0.08 | 0 | 0 - 0 |
| Northeast | 482 | 0.2 (0.2, 0.2) | ---^a^ | ---^a^ | ---^a^ | ---^a^ | ---^a^ |
| South | 716 | 0.2 (0.1, 0.2) | 45 | 0 (0,0) | 0.06 | 0 | 0 - 0 |
| West | 544 | 0.2 (0.2, 0.2) | 7 | 0 (0,0) | 0.06 | 0 | 0 - 0 |
| Missing | 56 | 0.2 (0.1, 0.2) | 0 | 0 (0,0) | 0.06 | 0 | |

^a^Data suppressed due to small cell counts (n<5)
