# Supplementary material for: Comparative analysis of influenza healthcare disparities in the United States using retrospective administrative claims from Medicaid and commercial databases, 2015–2019
Source: PLoS One. 2025 May 22;20(5):e0321208. doi: 10.1371/journal.pone.0321208 (PMC12097570; doi:10.1371/journal.pone.0321208)
Supplement: S5 Table — (DOCX) [file pone.0321208.s005.docx]

S5 Table: Influenza Antigen Testing by Sex, Race/Ethnicity, US Region, and Index Setting

|  |  |  | 18-64 years | | | | |
| --- | --- | --- | --- | --- | --- | --- | --- |
|  | Medicaid | | CDM | |  | | |
|  | **#** | **Proportion (95% CI)** | **#** | **Proportion (95% CI)** | **SMD** | **RR** | **CI** |
| None |  |  |  |  |  | |  |
| SEX |  |  |  |  |  | |  |
| Female | 533013 | 59.6 (59.5, 59.7) | 99837 | 40.1 (39.9, 40.3) | 0.4 | 1.48 | 1.47 - 1.49 |
| Male | 230903 | 61.9 (61.8, 62.1) | 81077 | 41.2 (40.9, 41.5) | 0.42 | 0.67 | 0.665 - 0.675 |
| RACE |  |  |  |  |  | |  |
| Asian | 49188 | 70.4 (70.1, 70.8) | 9611 | 47 (46.1, 47.9) | 0.49 | 1.5 | 1.468 - 1.533 |
| Black | 153941 | 65.4 (65.2, 65.6) | 17624 | 39.6 (39, 40.2) | 0.53 | 1.65 | 1.624 - 1.676 |
| Hispanic | 129789 | 66.7 (66.5, 66.9) | 24031 | 43.2 (42.7, 43.7) | 0.49 | 1.54 | 1.519 - 1.561 |
| White | 313444 | 58 (57.9, 58.1) | 122148 | 39.8 (39.6, 40) | 0.37 | 1.46 | 1.45 - 1.47 |
| Missing | 115954 | 51.7 (51.4, 51.9) | 7819 | 43.7 (42.8, 44.6) | 0.16 | 1.18 | 1.153 - 1.207 |
| Other | 1612 | 56.1 (54.3, 57.9) |  |  |  | |  |
| REGION |  |  |  |  |  | |  |
| Midwest | 164680 | 62.9 (62.7, 63) | 37657 | 42.9 (42.5, 43.3) | 0.41 | 1.47 | 1.454 - 1.487 |
| Northeast | 161435 | 71.5 (71.3, 71.7) | 22715 | 60 (59.2, 60.8) | 0.24 | 1.19 | 1.174 - 1.207 |
| South | 208621 | 45.8 (45.7, 46) | 83396 | 32.3 (32.1, 32.5) | 0.28 | 1.42 | 1.409 - 1.431 |
| West | 205258 | 70.3 (70.1, 70.5) | 36125 | 59.3 (58.7, 59.9) | 0.23 | 1.18 | 1.167 - 1.193 |
| Missing | 23934 | 73.2 (72.7, 73.7) | 363 | 49.9 (45.2, 54.6) | 0.49 | 1.47 | 1.325 - 1.631 |
| INDEX SETTING |  |  |  |  |  | |  |
| Emergency Department | 395345 | 65 (64.9, 65.1) | 21520 | 45.2 (44.6, 45.8) | 0.41 | 1.44 | 1.42 - 1.46 |
| Intensive Care Unit | 2496 | 93.5 (92.5, 94.4) | 68 | 81.9 (62.6, 101.2) | 0.36 | 1.14 | 0.896 - 1.451 |
| Inpatient | 23229 | 94.4 (94.1, 94.7) | 3221 | 91.8 (88.7, 94.9) | 0.1 | 1.03 | 0.993 - 1.069 |
| Outpatient | 342858 | 54.2 (54.1, 54.4) | 153938 | 39 (38.9, 39.1) | 0.31 | 1.39 | 1.382 - 1.398 |
| Within 2 days |  |  |  |  |  | |  |
| SEX |  |  |  |  |  | |  |
| Female | 357236 | 39.9 (39.8, 40) | 142471 | 57.3 (57, 57.6) | -0.35 | 0.7 | 0.696 - 0.704 |
| Male | 140318 | 37.6 (37.5, 37.8) | 112308 | 57.1 (56.8, 57.4) | -0.4 | 0.66 | 0.655 - 0.665 |
| RACE |  |  |  |  |  | |  |
| Asian | 20403 | 29.2 (28.9, 29.6) | 10554 | 51.6 (50.6, 52.6) | -0.47 | 0.57 | 0.557 - 0.584 |
| Black | 80398 | 34.2 (34, 34.3) | 25712 | 57.8 (57.1, 58.5) | -0.49 | 0.59 | 0.582 - 0.598 |
| Hispanic | 63799 | 32.8 (32.6, 33) | 30361 | 54.6 (54, 55.2) | -0.45 | 0.6 | 0.592 - 0.608 |
| White | 224320 | 41.5 (41.4, 41.6) | 178335 | 58.1 (57.9, 58.3) | -0.34 | 0.71 | 0.706 - 0.714 |
| Missing | 107388 | 47.8 (47.6, 48) | 9672 | 54.1 (53.1, 55.1) | -0.13 | 0.88 | 0.862 - 0.899 |
| Other | 1248 | 43.5 (41.7, 45.3) |  |  |  | |  |
| REGION |  |  |  |  |  | |  |
| Midwest | 96144 | 36.7 (36.5, 36.9) | 48984 | 55.8 (55.3, 56.3) | -0.39 | 0.66 | 0.653 - 0.667 |
| Northeast | 63492 | 28.1 (27.9, 28.3) | 14717 | 38.9 (38.3, 39.5) | -0.23 | 0.72 | 0.707 - 0.733 |
| South | 243984 | 53.6 (53.4, 53.7) | 167439 | 64.9 (64.6, 65.2) | -0.23 | 0.83 | 0.825 - 0.835 |
| West | 85375 | 29.2 (29.1, 29.4) | 24120 | 39.6 (39.1, 40.1) | -0.22 | 0.74 | 0.729 - 0.751 |
| Missing | 8561 | 26.2 (25.7, 26.7) | 357 | 49.1 (44.7, 53.5) | -0.49 | 0.53 | 0.477 - 0.589 |
| INDEX SETTING |  |  |  |  |  | |  |
| Emergency Department | 209969 | 34.5 (34.4, 34.6) | 24923 | 52.4 (51.8, 53) | -0.37 | 0.66 | 0.651 - 0.669 |
| Intensive Care Unit | 158 | 5.9 (5.1, 6.9) | 10 | 12 (4.9, 19.1) | -0.22 | 0.49 | 0.259 - 0.928 |
| Inpatient | 1227 | 5 (4.7, 5.3) | 259 | 7.4 (6.5, 8.3) | -0.1 | 0.68 | 0.595 - 0.778 |
| Outpatient | 286202 | 45.3 (45.2, 45.4) | 231966 | 58.8 (58.6, 59) | -0.27 | 0.77 | 0.766 - 0.774 |
| 3 to 28 days |  |  |  |  |  | |  |
| SEX |  |  |  |  |  | |  |
| Female | 4605 | 0.5 (0.5, 0.5) | 6391 | 2.6 (2.5, 2.7) | -0.17 | 0.19 | 0.183 - 0.197 |
| Male | 1545 | 0.4 (0.4, 0.4) | 3313 | 1.7 (1.6, 1.8) | -0.13 | 0.24 | 0.226 - 0.255 |
| RACE |  |  |  |  |  | |  |
| Asian | 236 | 0.3 (0.3, 0.4) | 298 | 1.5 (1.4, 1.6) | -0.12 | 0.21 | 0.177 - 0.249 |
| Black | 1049 | 0.4 (0.4, 0.5) | 1113 | 2.5 (2.3, 2.7) | -0.18 | 0.16 | 0.147 - 0.174 |
| Hispanic | 1013 | 0.5 (0.5, 0.6) | 1226 | 2.2 (2.1, 2.3) | -0.15 | 0.23 | 0.212 - 0.25 |
| White | 2689 | 0.5 (0.5, 0.5) | 6565 | 2.1 (2.1, 2.1) | -0.14 | 0.23 | 0.22 - 0.241 |
| Missing | 1151 | 0.5 (0.5, 0.5) | 388 | 2.2 (2, 2.4) | -0.15 | 0.23 | 0.205 - 0.258 |
| Other | 12 | 0.4 (0.2, 0.7) |  |  |  | |  |
| REGION |  |  |  |  |  | |  |
| Midwest | 1150 | 0.4 (0.4, 0.5) | 1171 | 1.3 (1.2, 1.4) | -0.1 | 0.3 | 0.277 - 0.325 |
| Northeast | 801 | 0.4 (0.3, 0.4) | 446 | 1.2 (1.1, 1.3) | -0.09 | 0.34 | 0.303 - 0.382 |
| South | 2695 | 0.6 (0.6, 0.6) | 7327 | 2.8 (2.7, 2.9) | -0.17 | 0.21 | 0.201 - 0.219 |
| West | 1295 | 0.4 (0.4, 0.5) | 633 | 1 (0.9, 1.1) | -0.08 | 0.38 | 0.346 - 0.418 |
| Missing | 209 | 0.6 (0.6, 0.7) | 7 | 1 (0.4, 1.6) | -0.04 | 0.62 | 0.292 - 1.317 |
| INDEX SETTING |  |  |  |  |  | |  |
| Emergency Department | 2877 | 0.5 (0.5, 0.5) | 1161 | 2.4 (2.3, 2.5) | -0.16 | 0.21 | 0.196 - 0.225 |
| Intensive Care Unit | 16 | 0.6 (0.4, 1) | ---^a^ | ---^a^ | ---^a^ | ---^a^ | ---^a^ |
| Inpatient | 155 | 0.6 (0.5, 0.7) | 28 | 0.8 (0.5, 1.1) | -0.02 | 0.75 | 0.501 - 1.122 |
| Outpatient | 3102 | 0.5 (0.5, 0.5) | 8358 | 2.1 (2.1, 2.1) | -0.14 | 0.24 | 0.23 - 0.25 |

^a^Data suppressed due to small cell counts (n<5)
