# Supplementary material for: Comparative analysis of influenza healthcare disparities in the United States using retrospective administrative claims from Medicaid and commercial databases, 2015–2019
Source: PLoS One. 2025 May 22;20(5):e0321208. doi: 10.1371/journal.pone.0321208 (PMC12097570; doi:10.1371/journal.pone.0321208)
Supplement: S6 Table — (DOCX) [file pone.0321208.s006.docx]

S6 Table: Proportion of Influenza RT-PCR By Sex, Race/Ethnicity, US Region, and Index Setting

|  |  |  |  |  |  |  |  |
| --- | --- | --- | --- | --- | --- | --- | --- |
|  | Medicaid | | CDM | |  | | |
|  | **#** | **Proportion (95% CI)** | **#** | **Proportion (95% CI)** | **SMD** | **RR** | **CI** |
| None | | | | | | | |
| SEX |  |  |  |  |  | |  |
| Female | 802809 | 89.7 (89.7, 89.8) | 235472 | 94.7 (94.5, 94.9) | -0.19 | 0.95 | 0.946 - 0.954 |
| Male | 331610 | 89 (88.9, 89.1) | 186133 | 94.6 (94.3, 94.9) | -0.21 | 1.06 | 1.054 - 1.066 |
| RACE |  |  |  |  |  | |  |
| Asian | 62713 | 89.8 (89.6, 90) | 19402 | 94.8 (93.9, 95.7) | -0.19 | 0.95 | 0.935 - 0.965 |
| Black | 206164 | 87.6 (87.5, 87.7) | 41630 | 93.7 (93.1, 94.3) | -0.21 | 0.94 | 0.93 - 0.95 |
| Hispanic | 176334 | 90.6 (90.5, 90.7) | 53463 | 96.1 (95.6, 96.6) | -0.22 | 0.94 | 0.931 - 0.949 |
| White | 480852 | 89 (88.9, 89.1) | 290401 | 94.6 (94.4, 94.8) | -0.2 | 0.94 | 0.936 - 0.944 |
| Missing | 205875 | 91.7 (91.6, 91.8) | 16764 | 93.8 (92.9, 94.7) | -0.08 | 0.98 | 0.965 - 0.996 |
| Other | 2489 | 86.7 (85.4, 87.9) |  |  |  | |  |
| REGION |  |  |  |  |  | |  |
| Midwest | 216656 | 82.7 (82.6, 82.8) | 78949 | 89.9 (89.5, 90.3) | -0.21 | 0.92 | 0.913 - 0.928 |
| Northeast | 211656 | 93.8 (93.7, 93.9) | 35238 | 93 (92.2, 93.8) | 0.03 | 1.01 | 0.999 - 1.021 |
| South | 420968 | 92.5 (92.4, 92.5) | 249508 | 96.6 (96.4, 96.8) | -0.18 | 0.96 | 0.955 - 0.965 |
| West | 254471 | 87.2 (87, 87.3) | 57277 | 94.1 (93.5, 94.7) | -0.24 | 0.93 | 0.922 - 0.938 |
| Missing | 30676 | 93.8 (93.5, 94.1) | 697 | 95.9 (91.2, 100.6) | -0.1 | 0.98 | 0.909 - 1.056 |
| INDEX SETTING |  |  |  |  |  | |  |
| Emergency Department | 509225 | 83.7 (83.6, 83.8) | 40872 | 85.9 (85.3, 86.5) | -0.06 | 0.97 | 0.96 - 0.98 |
| Intensive Care Unit | 2597 | 97.3 (96.6, 97.8) | 74 | 89.3 (70, 108.6) | 0.32 | 1.09 | 0.865 - 1.373 |
| Inpatient | 23930 | 97.2 (97, 97.4) | 3457 | 98.5 (95.4, 101.6) | -0.09 | 0.99 | 0.955 - 1.026 |
| Outpatient | 598675 | 94.7 (94.6, 94.8) | 377283 | 95.7 (95.6, 95.8) | -0.05 | 0.99 | 0.986 - 0.994 |
| Within 2 days | | | | | | | |
| SEX |  |  |  |  |  | |  |
| Female | 90603 | 10.1 (10.1, 10.2) | 12670 | 5.1 (4.8, 5.4) | 0.19 | 1.98 | 1.944 - 2.017 |
| Male | 40569 | 10.9 (10.8, 11) | 10203 | 5.2 (4.9, 5.5) | 0.21 | 2.1 | 2.055 - 2.146 |
| RACE |  |  |  |  |  | |  |
| Asian | 6995 | 10 (9.8, 10.2) | 1025 | 5 (4, 6) | 0.19 | 2 | 1.873 - 2.136 |
| Black | 28842 | 12.3 (12.1, 12.4) | 2693 | 6.1 (5.4, 6.8) | 0.22 | 2.03 | 1.951 - 2.112 |
| Hispanic | 17964 | 9.2 (9.1, 9.4) | 2069 | 3.7 (3.1, 4.3) | 0.22 | 2.47 | 2.36 - 2.585 |
| White | 58654 | 10.9 (10.8, 10.9) | 16015 | 5.2 (5, 5.4) | 0.21 | 2.09 | 2.054 - 2.127 |
| Missing | 18346 | 8.2 (8.1, 8.3) | 1078 | 6 (5, 7) | 0.08 | 1.36 | 1.279 - 1.446 |
| Other | 377 | 13.1 (11.9, 14.4) |  |  |  | |  |
| REGION |  |  |  |  |  | |  |
| Midwest | 44674 | 17.1 (16.9, 17.2) | 8616 | 9.8 (9.3, 10.3) | 0.21 | 1.74 | 1.7 - 1.781 |
| Northeast | 13868 | 6.1 (6, 6.2) | 2497 | 6.6 (6, 7.2) | -0.02 | 0.93 | 0.891 - 0.97 |
| South | 33870 | 7.4 (7.4, 7.5) | 8256 | 3.2 (2.9, 3.5) | 0.19 | 2.31 | 2.255 - 2.366 |
| West | 36780 | 12.6 (12.5, 12.7) | 3471 | 5.7 (5.2, 6.2) | 0.24 | 2.21 | 2.134 - 2.288 |
| Missing | 1986 | 6.1 (5.8, 6.3) | 30 | 4.1 (-0.3, 8.5) | 0.09 | 1.5 | 1.043 - 2.157 |
| INDEX SETTING |  |  |  |  |  | |  |
| Emergency Department | 97930 | 16.1 (16, 16.2) | 6622 | 13.9 (13.3, 14.5) | 0.06 | 1.16 | 1.131 - 1.189 |
| Intensive Care Unit | 61 | 2.3 (1.8, 2.9) | ---^a^ | ---^a^ | ---^a^ | ---^a^ | ---^a^ |
| Inpatient | 570 | 2.3 (2.1, 2.5) | 43 | 1.2 (0.3, 2.1) | 0.08 | 1.88 | 1.379 - 2.564 |
| Outpatient | 32617 | 5.2 (5.1, 5.2) | 16181 | 4.1 (3.9, 4.3) | 0.05 | 1.27 | 1.246 - 1.294 |
| 3 to 28 days | | | | | | | |
| SEX |  |  |  |  |  | |  |
| Female | 1442 | 0.2 (0.2, 0.2) | 557 | 0.2 (0.1, 0.3) | -0.01 | 0.89 | 0.807 - 0.981 |
| Male | 587 | 0.2 (0.1, 0.2) | 362 | 0.2 (0.1, 0.3) | 0 | 1.09 | 0.956 - 1.243 |
| RACE |  |  |  |  |  | |  |
| Asian | 119 | 0.2 (0.1, 0.2) | 36 | 0.2 (0.1, 0.3) | 0.01 | 1.14 | 0.785 - 1.656 |
| Black | 382 | 0.2 (0.1, 0.2) | 126 | 0.3 (0.1, 0.5) | -0.02 | 0.7 | 0.572 - 0.856 |
| Hispanic | 303 | 0.2 (0.1, 0.2) | 87 | 0.2 (0.1, 0.3) | 0.01 | 1.28 | 1.008 - 1.625 |
| White | 947 | 0.2 (0.2, 0.2) | 632 | 0.2 (0.2, 0.2) | 0 | 0.97 | 0.877 - 1.073 |
| Missing | 272 | 0.1 (0.1, 0.1) | 38 | 0.2 (0, 0.4) | -0.03 | 0.47 | 0.334 - 0.661 |
| Other |  |  |  |  |  | |  |
| REGION |  |  |  |  |  | |  |
| Midwest | 644 | 0.2 (0.2, 0.3) | 247 | 0.3 (0.2, 0.4) | -0.02 | 0.71 | 0.613 - 0.822 |
| Northeast | 204 | 0.1 (0.1, 0.1) | 143 | 0.4 (0.3, 0.5) | -0.06 | 0.26 | 0.21 - 0.322 |
| South | 462 | 0.1 (0.1, 0.1) | 398 | 0.2 (0.1, 0.3) | -0.02 | 0.65 | 0.568 - 0.743 |
| West | 677 | 0.2 (0.2, 0.3) | 130 | 0.2 (0.1, 0.3) | 0 | 0.93 | 0.771 - 1.122 |
| Missing | 42 | 0.1 (0.1, 0.2) | 0 | 0 (0,0) | -0.04 | 0 | 0 - NaN |
| INDEX SETTING |  |  |  |  |  | |  |
| Emergency Department | 1036 | 0.2 (0.2, 0.2) | 111 | 0.2 (0.1, 0.3) | -0.01 | 0.86 | 0.707 - 1.046 |
| Intensive Care Unit | 12 | 0.4 (0.3, 0.8) | ---^a^ | ---^a^ | ---^a^ | ---^a^ | ---^a^ |
| Inpatient | 111 | 0.5 (0.4, 0.5) | 8 | 0.2 (-0.1, 0.5) | 0.05 | 2.2 | 1.073 - 4.51 |
| Outpatient | 870 | 0.1 (0.1, 0.1) | 798 | 0.2 (0.2, 0.2) | -0.03 | 0.49 | 0.445 - 0.539 |

^a^Data suppressed due to small cell counts (n<5)
